# The Relationship Between Math Anxiety and Math Performance: A Meta-Analytic Investigation

**DOI:** 10.3389/fpsyg.2019.01613

**Published:** 2019-08-07

**Authors:** Jing Zhang, Nan Zhao, Qi Ping Kong

**Affiliations:** ^1^Faculty of Education, College of Teacher Education, East China Normal University, Shanghai, China; ^2^School of Psychology and Cognitive Science, East China Normal University, Shanghai, China

**Keywords:** math anxiety, math performance, meta-analysis, students, moderator analysis

## Abstract

Math anxiety (MA) has been suggested to decrease the math performance of students. However, it remains unclear what factors moderate this relationship. The aim of this research was to explore the link between MA and math performance. Studies that explored the math anxiety-performance link, conducted from 2000 to 2019 (84 samples, *N* = 8680), were identified and statistically integrated with a meta-analysis method. The results indicated a robust negative math anxiety-performance link. Furthermore, regarding the analysis of moderator variables, this negative link was stronger in the studies that involved Asian students, but this link was the weakest in the studies that involved European students. Moreover, this negative link was stronger in the studies within a senior high school group, whereas it was the weakest in the studies within an elementary group. Finally, this negative link was strongest among studies that used a custom test and studies that assessed problem-solving skills. Potential explanations and implications for research and practice are discussed.

## Introduction

Math anxiety (MA) has been a matter of concern in education for a long time and refers to the state of fear, tension, and apprehension when individuals engage with math (Ashcraft, 2002; Ashcraft and Ridley, 2005). A range of studies suggested that this phenomenon is a highly prevalent problem among students from elementary schools to universities (Betz, 1978; Ma and Xu, 2004; Rodarte-Luna and Sherry, 2008; Jain and Dowson, 2009; Gunderson et al., 2018). The negative math anxiety-performance link has been found in many empirical studies, which indicates MA would lead to poor performance when individuals deal with math reasoning or solve math problems (Bandalos et al., 1995; Ashcraft and Kirk, 2001; Ashcraft, 2002; Cates and Rhymer, 2003; Ma and Xu, 2004; Miller and Bichsel, 2004). However, the effect sizes of these studies are variable according to different reasons; thus, a systematic analysis is necessary to understand the math anxiety-performance link and the role that moderators play.

There have been nearly 20,200 previous studies related to MA or math performance (Olmez and Ozel, 2012; Necka et al., 2015; Lukowski et al., 2016; Justicia-Galiano et al., 2017), while few systematic studies focus on exploring the math anxiety-performance link during the previous 19 years. Two previous meta-analyses that were conducted 30 or 40 years ago concluded that there was a small but robust negative math anxiety-performance link in students (Hembree, 1990; Ma, 1999). However, for Ma's review, only 26 studies were included for which students originated from elementary and secondary schools. For Hembree's review, the analysis did not focus on the math anxiety-performance link. Therefore, to guide future studies in math education, an up-to-date meta-analysis exploring the math anxiety-performance link in recent years is needed.

Furthermore, based on the studies from 2000 to 2018, a range of potential moderators, including gender, grade level, geographical region, measurement of math anxiety, measurements of math performance, measurement aspects of math performance and publication year, may influence the math anxiety-performance link (Yaratan and Kasapoglu, 2012; Vukovic et al., 2013a; Hill et al., 2016; Gunderson et al., 2018). Moreover, the results of the PISA also indicated that MA could be affected by a range of factors (Foley et al., 2017), such as variations in the methodology and the educational level of participants (e.g., elementary school, junior high school and senior high school) (Krinzinger et al., 2009; Passolunghi et al., 2014). Thus, we sought to examine whether these features moderate the math anxiety-performance link and how these factors moderate this association to provide guidance for future research. Collectively, it would be informative to consider these factors in our present meta-analysis.

### Gender

A range of research has shown that gender might modulate the math anxiety-performance link. First, gender has been suggested to modulate math anxiety (Mustafa and KoçAk, 2006); however, the findings were inconsistent. Several studies showed significantly stronger MA in females than in males (Osborne, 2001; Yüksel-Sahin, 2008; Dowker et al., 2012; Gunderson et al., 2018) For example, Maloney et al. (2012) suggested that women might have stronger MA than men when dealing with tasks that involve mathematical skills and numerical skills. Other findings suggested that girls had greater habitual MA than boys; however, they did not experience higher level MA than boys during math content learning or a math content test (Goetz et al., 2013). In light of these inconsistent findings, the effect of gender on the math anxiety-performance link should be explored.

### Grade Level

It is suggested that MA might impact performance over time (Ramirez et al., 2013; Vukovic et al., 2013a; Maloney et al., 2015) As the difficulty of math learning increases with age, the math anxiety might also increases (Olmez and Ozel, 2012). Recent studies indicated that a negative math anxiety-performance link existed for young adults (Wu et al., 2017; Gunderson et al., 2018). However, the relationship between MA and math performance remained unclear for the elementary group. Some indicated that MA of primary school students was not related to performance (Thomas and Dowker, 2000; Dowker et al., 2012; Wood et al., 2012; Haase et al., 2012), while other studies have suggested that the math anxiety-performance link existed even at this age (Wu et al., 2012, 2014; Jameson, 2013; Vukovic et al., 2013a). Based on these results, grade level might modulate the math anxiety-performance link.

### Geographical Region

Previous studies have implied that geographical region may influence the math anxiety-performance link. For example, the negative math anxiety-performance link was stronger in China than in the USA according to the findings of Ching (2017) and Wu et al. (2017). Ching (2017) found a negative link (*r* = −0.318) between MA and math performance in young Chinese students. Moreover, Wu et al. (2017) also indicated a negative link (*r* = −0.23) between MA and math performance in young USA children. In Asia, academic achievement is highly valued, which would lead to the high-level anxiety of Asian students (Ho et al., 2000). In contrast, students seem to be less critical of their academic performance and feel relaxed in European countries. This relationship was also examined by several studies that compared one or two countries (e.g., American, Chinese, and Taiwanese in Ho et al., 2000; Arab and Israeli in Birenbaum et al., 2005). However, no consistent results have been found, which made it difficult to claim a general pattern of this link across cultures. Therefore, additional details regarding how cross-cultural information modulated this negative relationship are needed.

### Measurement of Math Performance

Recent studies have suggested that the measurement of different aspects of math performance had variable effects on the correlation between MA and math performance (Ramirez et al., 2016; Lee and Cho, 2018). For example, Beilock and Willingham (2014) found a strongly math anxiety-performance link especially in mathematical reasoning, which assessed advanced problem-solving skills. In contrast, Harari et al. (2013) reported a weak link between MA and digital calculation, which assessed basic computational skills. In terms of the measurement form, both standard tests (e.g., Henschel and Roick, 2017; Wu et al., 2017) and custom assessments (e.g., Necka et al., 2015; Ganley and Mcgraw, 2016) were used to estimate math performance, which have not previously been noted. Overall, the incorporation of the measurement aspects and forms of math performance as two potential moderator variables (measurement aspects of math performance vs. measurement forms of math performance) in the present study was necessary.

### Measurement of MA

In light of a range of scales used to measure MA, the variation in optimizing MA might be related to the variation in research findings. Specifically, some studies employed the Mathematics Anxiety Questionnaire (MAQ) to measure MA (Carey et al., 2017), which found no correlation between MA and math performance (Dowker et al., 2012; Wood et al., 2012; Haase et al., 2012). In contrast, some studies employed other questionnaires (e.g., a scale for earlsy MA, The Mathematical Anxiety for Young Children) to measure MA, which showed a negative relationship between MA and math performance (Wu et al., 2012, 2014). Thus, an assessment of whether the measurement of MA could modulate the math anxiety-performance link was also necessary.

### Publication Year

Several recent studies found no relationship between MA and math performance (Thomas and Dowker, 2000; Krinzinger et al., 2007, 2009; Harari et al., 2013; Vukovic et al., 2013a; Cargnelutti et al., 2017). For example, Cargnelutti et al. (2017) found no significant relationship (*r* = 0.04) between math performance and MA in 2nd graders. As educators and researchers have highlighted the importance of relieving the negative effect of MA on math performance, the relationship between MA and math performance might have decreased in recent years. Thus, the publication year should be considered.

### Study Purpose

Taken together, the present meta-analysis aimed to quantitatively synthesize these studies to provide an updated and overall view on the math anxiety-performance relationship and investigate the specific variables that may play a role in inconsistencies. First, we calculated the overall effect size of the correlations between MA and math performance. We then examined whether this correlation differed across gender, grade level, geographical regions, measurement of MA, measurement aspects of math performance, measurement forms of math performance and publication year.

## Methods

### Literature Search

We conducted a literature search for studies on MA and math performance from January 2000 to December 2018 using electronic databases, including Google Scholar, ERIC, EBSCO, Web of Science, ProQuest, PsycINFO, PsycARTICLES, and PsycCRITIQUES. The following key descriptions were used to search for related articles: “mathematics (math) anxiety,” “mathematics(math) performance,” “mathematics(math) outcome,” “mathematics(math) achievement”) “math anxiety” AND “math performance.” We then refined the search following inclusion criteria.

### Literature Inclusion Criteria

The articles were further screened based on the following criteria:

- MA and math achievement were both measured, and the results were both reported. Each study had to report the relationship between MA and math performance; Studies that only measure MA or only measure math performance were excluded. For example, Olmez and Ozel (2012) only explored MA among sixth and seventh grade Turkish elementary school students and did not investigate further to provide information regarding math performance.
- Individual studies should explicitly report the sample size; if studies did not report the sample size (e.g., Lee, 2009), they were all excluded.
- Studies that applied an eligible test method to assess MA and math performance were included in the analysis. Review studies were excluded. For example, Dowker et al. (2016) reviewed what research has indicated regarding MA in the last 60 years; Foley et al. (2017) systematically clarified the relationship between MA and math performance based on data from the Program for International Student Assessment (PISA). Although they clarified the relationship between MA and math performance, they did not conduct an experiment to adopt data. As a result, they were excluded.
- Each study should clearly report the Pearson correlation r or F and t values that could be transformed into r. Studies that only reported the results from regression modeling or multilevel modeling analysis (e.g., Nasser and Birenbaum, 2005; Shores and Shannon, 2007), which did not provide sufficient statistical information that could be transformed into r, were excluded.
- Each study should be published in a peer-reviewed journal written in English between 2000 and 2018. Studies that did not meet this criterion were excluded, for example, a study conducted in 1990 (Baya'a, 1990); moreover, non-English studies were not considered in our study.
- The grade level of participants in studies should be elementary school to university level; studies that focus on preschool students were excluded. For example, Aslan et al. (2013) explored the link between the MA of teachers and math performance of preschool students. These studies were excluded.

**Figure 1:**
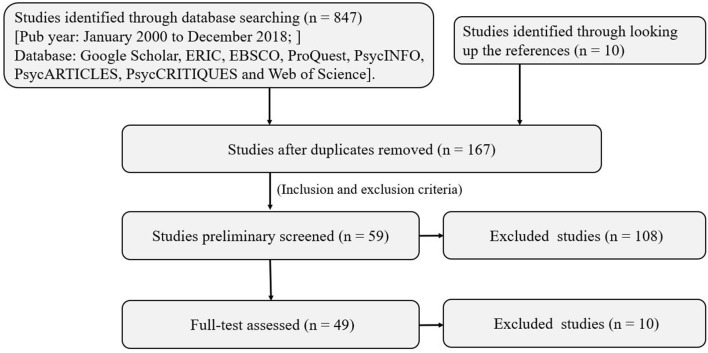
Literature search process.

Finally, 49 studies, including 84 independent samples, were included for analysis (Figure 1), as more than one independent sample was included in several studies. Studies that were not consistent with these criteria were excluded from this meta-analysis.

### Coding

To exclude the apparently ineligible studies (e.g., studies in non-English languages), two independent coders screened all studies from title to abstract. Relevant studies were then coded by coders independently at the full-text level to identify variable effect sizes and moderators. Moreover, to identify the potential moderators, a systematic process was employed.

Four categories were coded: (a) study information, which was organized in two categories, including publication year and geographical region (i.e., US, Asia, Europe, and other regions), (b) participant information, including number of total sample size, grade level and gender, and (c) methodology, which was organized in three categories: measurement of MA, measurement forms of math performance, and measurement aspects of math performance. We obtained relevant information regarding the previously described moderator variables through reading the research method sections of each individual studies. And then we got information about the previously described moderator variables. Table 1 showed the final list of study features.

**Table 1:** Coded study features as potential moderators.

**Moderator variable**
**Gender**
Boy
Girl
All
**Publication year**
Continuous: 2000-2018
**Grade level**
Elementary
Junior high school
Senior high school
Mixed
University
**Geographical region**
USA
Europe
Asia
Other
**Measure of MA**
AMAS (Abbreviated Math Anxiety Scale)
CMAQ (Child Math Anxiety Questionnaire)
MAQ (The Mathematics Anxiety Questionnaire)
MARS (Mathematics Anxiety Rating Scale)
MAS (Mathematics Anxiety Scale)
MASC (Mathematics Anxiety Scale for Children)
MASYC (Mathematics Anxiety Scale for Young Children)
SEMA (The Scale for Early Math Anxiety)
Other
**Measure of math performance**
Custom test
Standardized test
Other
**Measure aspects of math performance**
Computation
Problem solving
Whole

Two coders were enrolled to code all studies independently, according to the guidelines (Cooper, 2016), following the inclusion criteria previously described. The coders were all trained well and completely understood all coding criteria. To calculate the interrater reliability, coding discrepancies were identified on six original studies, indicating an agreement of 94.91% [i.e., (59–3)/59 = 94.91%] between two coders. Finally, a consensus was reached for disagreements between two coders after discussion at the end of the coding procedure.

### Data Analysis

#### Effect Size Calculation

Pearson's *r* as an index of the effect size for our meta-analysis was employed (refer to Mcgrath and Meyer, 2006; Fritz et al., 2012, given the benefits of using *r* as an effect size metric). Some studies did not report the Pearson's *r* directly; thus, we transferred inference test statistics (*t*-value, *F*-value, or chi-square) in those studies to Pearson's *r*. If only individual studies statistically significant, the conservative effect size was obtained, assuming a *p*-value of 0.05. According to Cooper et al. (2009), to ensure the stabilization of the sample distribution, we applied Fisher's *z* transformations. Then these values were transformed back into correlations, with effect sizes and confidence intervals. Thus, each *r* would go through the Fisher *z* transformation:

$$\begin{array}{r} \text{z}=\frac{1}{2}•\text{ln}\left(\frac{\text{r}+\text{1}}{\text{r}-\text{1}}\right) \end{array}$$

In the present study, we applied the random-effects model to calculate effects. The random-effects model assumed that the effect sizes of different samples/studies may come from different populations and different populations had its own sampling distribution (Borenstein et al., 2009; Card, 2011). Taking into account the effects of sample levels, our systematic analysis of potential moderate variables was calculating the random effects at sample levels. The diversity of experimental settings of individual studies was included in our study (e.g., gender, grades, and geographical region), which made a random-effect model an appropriate approach (Cooper, 2016). Moreover, Comprehensive Meta-Analysis (CMA, Version 2.2) was applied to conduct all necessary computations and analyses.

#### Moderator Analyses

The *Q* test (a heterogeneity test) was employed which refered to the variation between the research results in different studies. A significant *Q* statistic indicated that individual studies were not come from a common population. This calculation we referred to were all conducted by the CMA.

#### Evaluation of Publication Bias

Three approaches to explore publication bias were adopted in the study. The first approach was a funnel plot, which clearly presented all effect sizes (Card, 2011). The second method was the fail-safe *N* (e.g., classic fail-safe *N*), which estimated the number of studies with non-significant results (unpublished data) needed to cause the mean ES to become statistically non-significant (Rosenthal, 1979). The third method was the Egger's test (Egger, 1997), which was a linear regression method that assessed the publication bias by the funnel plot.

## Results

### Overview of Primary Studies

After the literature filtering, 49 articles yielding 84 independent samples were included in the present meta-analyses. Table 2 presented study name ES (effect size), N (sample size), gender, grade level and publication year. Table 3 reported measure of MA, measure of math performance, measure aspects of math and geographical region. Table 4 showed the descriptive information of the moderator variables. For the features of the participants: 86% of studies did not analyze the effect of gender differences in the math anxiety-performance link, and only 14% of the reports consider the influence of gender on this link. Second, regarding the grade level of the participants, elementary students account for the largest proportion (53%), while 25% were university students, and few were junior or senior high school students. Furthermore, considering the regions where the primary study was conducted, studies conducted in Europe accounted for the largest proportion (37%). With respect to measurements of MA, the most adopted was the MARS (Mathematics Anxiety Rating Scale) (31%), followed by other scales (21%) and the AMAS (Abbreviated Math Anxiety Scale) (16%). Finally, considering the measurement forms of math performance, the largest proportion of studies applied standardized tests (61%), followed by a custom test (33%) and other forms (6%). In terms of the measurement aspects of math performance, the largest proportion of studies estimated general skills (47%), followed by the assessment of computation (35%) and problem-solving (8%).

**Table 2:** Summary of studies included in the meta-analysis (1).

**Number**	**Study name**	**ES**	***N***	**Gender**	**Grade level**	**Publication year**
1	Abu-Hilal, 2000	−0.360	394	Both	Elementary	2000
2	Ashcraft and Kirk, 2001①	−0.290	66	Both	University	2001
3	Ashcraft and Kirk, 2001②	−0.350	45	Both	University	2001
4	Ashcraft and Kirk, 2001③	−0.670	45	Both	University	2001
5	Ashkenazi and Danan, 2017	−0.540	58	Both	University	2017
6	Birgin et al., 2010①	−0.690	74	Both	Junior high school	2010
7	Birgin et al., 2010②	−0.720	73	Both	Junior high school	2010
8	Birgin et al., 2010③	−0.750	73	Both	Junior high school	2010
9	Brunyé et al., 2013	−0.420	36	Both	University	2013
10	Buelow and Frakey, 2013	−0.227	172	Both	University	2013
11	Cargnelutti et al., 2017①	−0.010	118	Both	Elementary	2017
12	Cargnelutti et al., 2017②	−0.310	80	Both	Elementary	2017
13	Ching, 2017①	−0.318	246	Both	Elementary	2017
14	Ching, 2017②	−0.216	246	Both	Elementary	2017
15	Daneshamooz et al., 2012	−0.246	42	Both	University	2012
16	Devine et al., 2012①	−0.180	268	Boys	Junior high school	2012
17	Devine et al., 2012②	−0.349	165	Girls	Junior high school	2012
18	Dowker et al., 2012	−0.069	89	Both	Elementary	2012
19	Erturan and Jansen, 2015	−0.150	73	Boys	Mixed	2015
20	Erturan and Jansen, 2015	−0.370	61	Girls	Mixed	2015
21	Galla and Wood, 2012	−0.270	139	Both	Elementary	2012
22	Ganley and Mcgraw, 2016	−0.140	296	Both	Elementary	2016
23	Gunderson et al., 2018	−0.290	580	Both	Elementary	2018
24	Harari et al., 2013①	−0.300	106	Both	Elementary	2013
25	Henschel and Roick, 2017	−0.520	368	Both	Elementary	2017
26	Hill et al., 2016①	−0.070	317	Boys	Elementary	2016
27	Hill et al., 2016②	−0.130	322	Girls	Elementary	2016
28	Hill et al., 2016③	−0.280	194	Boys	Elementary	2016
29	Hill et al., 2016④	−0.340	148	Girls	Elementary	2016
30	Ho et al., 2000①	−0.446	211	Both	Elementary	2000
31	Ho et al., 2000②	−0.405	214	Both	Elementary	2000
32	Ho et al., 2000③	−0.317	246	Both	Elementary	2000
33	Hoffman, 2010①	−0.380	296	Both	University	2010
34	Hoffman, 2010②	−0.330	244	Both	University	2010
35	Hoffman, 2010③	−0.330	296	Both	University	2010
36	Hunt et al., 2017	0.210	77	Both	Elementary	2017
37	Justicia-Galiano et al., 2017①	−0.260	167	Both	Elementary	2017
38	Justicia-Galiano et al., 2017②	−0.270	167	Both	Elementary	2017
39	Karasel et al., 2010	−0.280	134	Both	Elementary	2010
40	Karimi and Venkatesan, 2009	−0.150	284	Both	Junior high school	2009
41	Kyttälä and Björn, 2014①	−0.110	99	Both	Elementary	2014
42	Kyttälä and Björn, 2014②	−0.130	50	Boys	Junior high school	2014
43	Kyttälä and Björn, 2014③	−0.080	49	Girls	Junior high school	2014
44	Lauer et al., 2018	−0.190	394	Both	Elementary	2018
45	Lukowski et al., 2016	−0.260	244	Both	Elementary	2016
46	Luo et al., 2009	−0.389	311	Both	Junior high school	2009
47	Miller and Bichsel, 2004①	−0.320	100	Both	University	2004
48	Miller and Bichsel, 2004②	−0.410	100	Both	University	2004
49	Necka et al., 2015	−0.355	131	Both	University	2015
50	Núñez-Peña et al., 2013	−0.224	193	Both	University	2013
51	Olmez and Ozel, 2012①	−0.460	244	Both	Elementary	2012
52	Olmez and Ozel, 2012②	−0.370	77	Boys	Elementary	2012
53	Olmez and Ozel, 2012③	−0.580	72	Girls	Elementary	2012
54	Olmez and Ozel, 2012④	−0.670	75	Both	Elementary	2012
55	Olmez and Ozel, 2012	−0.280	74	Both	Elementary	2012
56	Passolunghi et al., 2016	−0.374	66	Both	Elementary	2016
57	Ramirez et al., 2016①	−0.360	304	Both	Elementary	2016
58	Ramirez et al., 2016②	−0.050	221	Both	Elementary	2016
59	Reali et al., 2016①	−0.080	136	Boys	Mixed	2016
60	Reali et al., 2016②	−0.240	160	Girls	Mixed	2016
61	Schillinger et al., 2018①	−0.270	341	Both	University	2018
62	Schillinger et al., 2018②	−0.410	341	Both	University	2018
63	Sherman and Wither, 2003①	−0.390	156	Both	Senior high school	2003
64	Sherman and Wither, 2003②	−0.318	298	Both	Junior high school	2003
65	Sherman and Wither, 2003③	−0.495	156	Both	Senior high school	2003
66	Sherman and Wither, 2003④	−0.461	156	Both	Junior high school	2003
67	Sherman and Wither, 2003	−0.423	156	Both	Junior high school	2003
68	Skaalvik, 2018	−0.330	939	Both	Junior high school	2018
69	Sorvo et al., 2017①	−0.050	295	Both	Elementary	2017
70	Sorvo et al., 2017②	−0.120	383	Both	Elementary	2017
71	Sorvo et al., 2017③	−0.090	178	Both	Elementary	2017
72	Sorvo et al., 2017④	−0.100	471	Both	Elementary	2017
73	Tsui et al., 2007	−0.447	36	Both	Elementary	2007
74	Vukovic et al., 2013b	−0.310	78	Both	Elementary	2013
75	Woodard, 2004	−0.200	125	Both	University	2004
76	Wu et al., 2012	−0.203	162	Both	Elementary	2012
77	Wu et al., 2014	−0.172	361	Both	University	2014
78	Wu et al., 2014	−0.380	366	Both	Elementary	2014
79	Wu et al., 2017①	−0.380	330	Both	Elementary	2017
80	Wu et al., 2017②	−0.230	330	Both	Elementary	2017
81	Yaratan and Kasapoglu, 2012	−0.591	188	Both	Junior high school	2012
82	Yüksel-Sahin, 2008	−0.207	249	Both	Elementary	2008
83	Zakaria and Nordin, 2008	−0.320	88	Both	University	2008
84	Zakaria et al., 2012	0.001	195	Both	Junior high school	2012

*We use first author and publication year to represent each study, and if one study provided more than one effect size, we use ①, ②, ③, ④, to indicate*.

*Effect size refers to Pearson's r in each study*.

**Table 3:** Summary of studies included in the meta-analysis (2).

**Number**	**Study name**	**Measure of MA**	**Measure of math performance**	**Measure aspects of math**	**Geographical region**
1	Abu-Hilal, 2000	Other	Custom test	Problem solving	Asia
2	Bandalos et al., 1995①	MARS	Custom test	Problem solving	USA
3	Bandalos et al., 1995②	MARS	Custom test	Problem solving	USA
4	Bandalos et al., 1995③	MARS	Custom test	Problem solving	USA
5	Ashkenazi and Danan, 2017	MARS	Custom test	Computation	Asia
6	Birgin et al., 2010①	Other	Other	Whole	Asia
7	Birgin et al., 2010②	Other	Other	Whole	Asia
8	Birgin et al., 2010③	Other	Other	Whole	Asia
9	Brunyé et al., 2013	AMAS	Standardized test	Computation	USA
10	Buelow and Frakey, 2013	AMAS	Standardized test	Whole	USA
11	Cargnelutti et al., 2017①	SEMA	Standardized test	Computation	Europe
12	Cargnelutti et al., 2017②	SEMA	Standardized test	Computation	Europe
13	Ching, 2017①	Other	Standardized test	Computation	Europe
14	Ching, 2017②	Other	Standardized test	Problem solving	Europe
15	Daneshamooz et al., 2012	MARS	Custom test	Whole	Asia
16	Devine et al., 2012①	AMAS	custom test	Computation	Europe
17	Devine et al., 2012②	AMAS	Custom test	Computation	Europe
18	Dowker et al., 2012③	Other	Custom test	Computation	Europe
19	Erturan and Jansen, 2015①	MASC	Standardized test	Computation	Europe
20	Erturan and Jansen, 2015②	MASC	Standardized test	Computation	Europe
21	Galla and Wood, 2012	MASC	Standardized test	Whole	USA
22	Ganley and Mcgraw, 2016	MASYC	Custom test	Whole	USA
23	Gunderson et al., 2018	CMAQ	Standardized test	Problem solving	Asia
24	Harari et al., 2013	MARS	Standardized test	Computation	USA
25	Henschel and Roick, 2017	MARS	Standardized test	Whole	Europe
26	Hill et al., 2016①	AMAS	Standardized test	Computation	Europe
27	Hill et al., 2016②	AMAS	Standardized test	Computation	Europe
28	Hill et al., 2016③	AMAS	Standardized test	Computation	Europe
29	Hill et al., 2016④	AMAS	Standardized test	Computation	Europe
30	Ho et al., 2000①	MAQ	Standardized test	Whole	Asia
31	Ho et al., 2000②	MAQ	Standardized test	Whole	Asia
32	Ho et al., 2000③	MAQ	Standardized test	Whole	USA
33	Hoffman, 2010①	MASYC	Custom test	Computation	USA
34	Hoffman, 2010②	MARS	Custom test	Computation	USA
35	Hoffman, 2010③	MASYC	Custom test	Computation	USA
36	Hunt et al., 2017	MASC	Custom test	Computation	Europe
37	Justicia-Galiano et al., 2017①	AMAS	Standardized test	Whole	Europe
38	Justicia-Galiano et al., 2017②	AMAS	Standardized test	Problem solving	Europe
39	Karasel et al., 2010	Other	Standardized test	Problem solving	Europe
40	Karimi and Venkatesan, 2009	MARS	Custom test	Whole	Asia
41	Kyttälä and Björn, 2014①	MARS	Standardized test	Computation	Europe
42	Kyttälä and Björn, 2014②	MARS	Standardized test	Computation	Europe
43	Kyttälä and Björn, 2014③	MARS	Standardized test	Computation	Europe
44	Lauer et al., 2018	AMAS	Custom test	Whole	USA
45	Lukowski et al., 2016	MARS	Standardized test	Computation	USA
46	Luo et al., 2009	MAQ	Other	Whole	Asia
47	Miller and Bichsel, 2004①	MARS	Standardized test	Problem solving	USA
48	Miller and Bichsel, 2004②	MARS	Standardized test	Computation	USA
49	Necka et al., 2015	MARS	Custom test	Computation	USA
50	Núñez-Peña et al., 2013	MARS	Custom test	Whole	Europe
51	Olmez and Ozel, 2012①	Other	Standardized test	Whole	Asia
52	Olmez and Ozel, 2012②	Other	Standardized test	Whole	Asia
53	Olmez and Ozel, 2012③	Other	Standardized test	Whole	Asia
54	Olmez and Ozel, 2012④	Other	Standardized test	Whole	Asia
55	Olmez and Ozel, 2012	Other	Standardized test	Whole	Asia
56	Passolunghi et al., 2016	AMAS	Standardized test	Whole	Europe
57	Ramirez et al., 2016①	CMAQ	Standardized test	Problem solving	USA
58	Ramirez et al., 2016②	CMAQ	Standardized test	Problem solving	USA
59	Reali et al., 2016	AMAS	Standardized test	Whole	Europe
60	Reali et al., 2016	AMAS	Standardized test	Whole	Europe
61	Schillinger et al., 2018①	MAS	Standardized test	Computation	Europe
62	Schillinger et al., 2018②	MAS	Standardized test	Whole	Europe
63	Sherman and Wither, 2003①	MARS	Custom test	Whole	Other
64	Sherman and Wither, 2003②	MARS	Custom test	Whole	Other
65	Sherman and Wither, 2003③	MARS	Custom test	Whole	Other
66	Sherman and Wither, 2003④	MARS	Custom test	Whole	Other
67	Sherman and Wither, 2003	MARS	Custom test	Whole	Other
68	Skaalvik, 2018	Other	Standardized test	Problem solving	Europe
69	Sorvo et al., 2017①	MAQ	Standardized test	Computation	Europe
70	Sorvo et al., 2017②	MAQ	Standardized test	Computation	Europe
71	Sorvo et al., 2017③	MAQ	Standardized test	Computation	Europe
72	Sorvo et al., 2017④	MAQ	Standardized test	Computation	Europe
73	Tsui et al., 2007	MARS	Standardized test	Computation	Europe
74	Vukovic et al., 2013b	MARS	Standardized test	Problem solving	USA
75	Woodard, 2004	MARS	Standardized test	Whole	USA
76	Wu et al., 2012	SEMA	Standardized test	Whole	USA
77	Wu et al., 2014	ESMA	Standardized test	Computation	USA
78	Wu et al., 2014	SEMA	Standardized test	Whole	USA
79	Wu et al., 2017①	Other	Standardized test	Problem solving	USA
80	Wu et al., 2017②	Other	Standardized test	Computation	USA
81	Yaratan and Kasapoglu, 2012	MAS	Custom test	Whole	other
82	Yüksel-Sahin, 2008	Other	Other	Whole	Asia
83	Zakaria and Nordin, 2008	MAS	Standardized test	Whole	Asia
84	Zakaria et al., 2012	FSMAS	Custom test	Whole	Asia

*We use first author and publication year to represent each study, and if one study provided more than one effect size, we use ①, ②, ③, ④, to indicate*.

**Table 4:** Descriptive statistics of included studies.

**Moderator variable**	**Identified categories**	**Counts(%)**
Gender	Boys or Girls	7(14)
	Both	42(86)
Grade level	Elementary	26(53)
	Junior high school	7(14)
	Senior high school	2(4)
	University	12(25)
	Mixed	2(4)
Geographical Region	USA	17(34)
	Europe	18(37)
	Asia	12(25)
	Other	2(4)
Measure of MA	AMAS	8(16)
	CMAQ	2(4)
	MAQ	3(6)
	MARS	15(31)
	MAS	3(6)
	MASC	3(6)
	MSYC	2(4)
	SEMA	3(6)
	Other	10(21)
Measure forms of math performance	Custom test	16(33)
	Standardized test	30(61)
	Other	3(6)
Measure aspects of math performance	Computation	17(35)
	Problem solving	9(18)
	Whole	23(47)

### Overall Analysis

Table 5 presents the major findings that resulted from the meta-analysis. The overall mean ES of the 49 articles was −0.3, with a 95% confidence interval that ranged from −0.35 to −0.28. Cohen (1988, 1992) suggested that ESs of 0.80, 0.50, and 0.20 presented large, medium, and small, respectively. Then, our findings suggested that there was a negative, although somehow weak, relationship between MA and mathematics performance.

**Table 5:** Relationship between mathematics anxiety and mathematics performance: overall results and moderator analyses.

**Moderators**	**Study-based meta-analysis**			
	***Q***	**k**	**Weighted *r***	**95% CI**
Overall	372.26	84	−0.32	(−0.35, −0.28)
Grade level	11.65			
Elementary		40	−0.27	(−0.32, −0.22)
Junior high school		19	−0.39	(−0.46, −0.32)
Senior high school		5	−0.44	(−0.55, −0.30)
Mixed		4	−0.21	(−0.38, −0.02)
University		16	−0.33	(−0.41, −0.24)
Gender	4.62			
Boy		7	−0.18	(−0.31, −0.04)
Girl		7	−0.30	(−0.43, −0.17)
All		70	−0.33	(−0.37, −0.29)
Geographical Region	29.60			
USA		20	−0.30	(−0.37, −0.22)
Europe		30	−0.21	(−0.27, −0.15)
Asia		23	−0.41	(−0.46, −0.35)
Other		11	−0.44	(−0.51, −0.35)
Measure of MA	15.62			
Abbreviated Math Anxiety Scale (AMAS)		13	−0.23	(−0.33, −0.14)
Child Math Anxiety Questionnaire (CMAQ)		3	−0.24	(−0.42, −0.04)
The Mathematics Anxiety Questionnaire (MAQ)		9	−0.28	(−0.38, −0.16)
Mathematics Anxiety Rating Scale (MARS)		30	−0.35	(−0.41, −0.29)
Mathematics Anxiety Scale (MAS)		3	−0.40	(−0.56, −0.21)
Mathematics Anxiety Scale for Children (MASC)		4	−0.15	(−0.34, −0.05)
Mathematics Anxiety Scale for Young Children (MASYC)		2	−0.26	(−0.48, −0.33)
The Scale for Early Math Anxiety (SEMA)		4	−0.24	(−0.40, −0.05)
Other		16	−0.41	(−0.50, −0.33)
Measure forms of math performance	13.02			
Custom test		30	−0.33	(−0.39, −0.27)
Standardized test		47	−0.28	(−0.33, −0.23)
Other		7	−0.51	(−0.60, −0.40)
Measure aspects of math performance	17.00			
Computation		28	−0.21	(−0.27, −0.15)
Problem solving		12	−0.33	(−0.42, −0.24)
Whole		44	−0.37	(−0.42, −0.33)

*Q* statistics indicated that the effect sizes were heterogeneous (*Q* = 526.50.10, *z* = −15.71, *p* < 0.001), which elucidated the differences of the ESs that were ascribed to sources. Thus, it also noted that the following-up analysis for potential moderator variables may reveal their contribution in this inconsistency.

**Figure 2:**
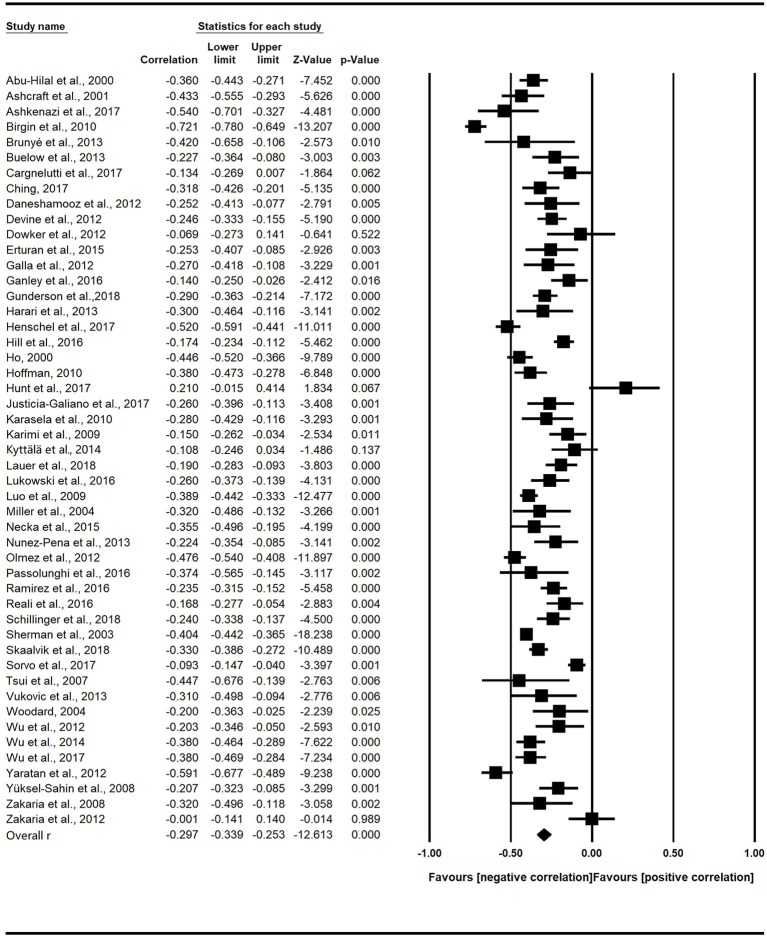
Forest plot.

The “forest plot” (Figure 2) presented the random-effects modeling analysis of the 49 studies and graphically showed the effect size (square dot) and its estimated confidence interval (horizontal lines extending from both sides of the squared dot). First, it was observed that only two effect sizes were positive (i.e., on the right side of the “null” effect line), e.g., Zakaria et al., 2012; Hunt et al., 2017), while the remaining effect sizes were negative. Second, it was clearly shown that the majority of the effect sizes had narrow confidence intervals, while several effect sizes had slightly wide confidence intervals (e.g., Tsui et al., 2007).

### Assessment of Publication Bias

**Figure 3:**
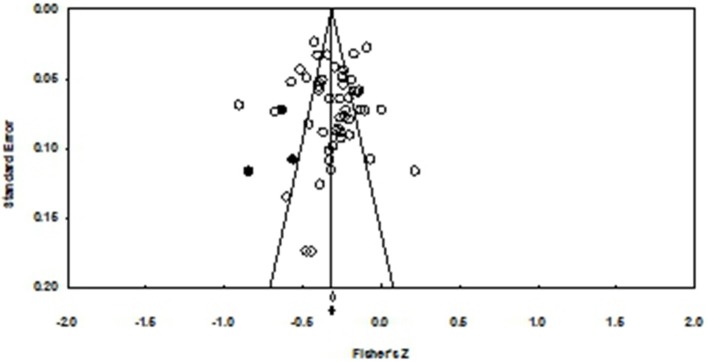
Funnel plot.

To estimate the publication bias, the funnel plot, Rosenthal's fail-safe N method and Egger test were all applied. First, the funnel plot was employed. Circles (referred to effect sizes) were symmetrically around the vertical line in the plot, which indicated no potential bias in our data (Figure 3). Second, Applied Rosenthal (1979) failsafe-N procedure, we obtained a value of 5535 missing studies at the *p*-value of 0.05. According to instruction of Rosenthal, when value of failsafe-N was larger than 5K + 10 (K represented the number of individual studies), we could safely refuse possible publication bias (Rothstein, 2008). In present research, 5K + 10 were 255 studies, which indicated our research have no publication bias. Egger's test (Egger, 1997) was also applied *t* = 0.19, *p* = 0.85, which suggested that the funnel plot was symmetric (Figure 3). Thus, we could safely eliminate the influence of publication bias on the validity of present research.

### Moderator Analysis

To test whether the math anxiety-performance link might be moderated by a range of variables (e.g., geographical region, measurement of MA, measurement of math performance, gender, grade level, and publication year), we conducted the heterogeneity test. The Q test (heterogeneity test) have found the differences among effect sizes, thus to explore which potential moderator (i.e., study features) might have played a role in these differences was needed.

### Geographical Region

Geographical regions were considered a potential moderate factor. The effect sizes were grouped into four broad groups: US, Europe, Asia and other counties. The statistical heterogeneity among the effect sizes was shown (*Q* = 29.60, *p* = 0.01). Specifically, the studies with Asian samples had the largest effect size (*r* = −0.41), while the studies with European samples showed the smallest effect size (*r* = −0.21). In addition, the studies with US samples had a larger effect size (*r* = −0.30) than the corresponding group in Europe, while they had a smaller effect size than the corresponding group in Asia.

### Gender

The moderate analyses on gender were conducted through two steps. First, the category on this variable was consistent with Ma's (1999) study, and we compared whether the math anxiety-performance link was significantly different among three categories (male, female, and mixed); the results suggested there were no differences among the three categories (*Q* = 4.62, *p* = 0.099). Second, this study tended to extend further to test when to exclude the confound effect from the mixed group and whether the average correlations between male and female were significantly different. Only 7 studies that provided the correlation between MA and math performance or necessary data could be transferred to effect sizes for different genders were included. The results suggested no significant difference between females (*r* = −0.30) and males (*r* = −0.18) (*Q* = 2.73, *p* = 0.098).

Table 5 Relationship between MA and math performance: overall results and moderator analyses.

### Grade Level

Five grade-level groups were formed in this meta-analysis, including an elementary group, junior high group, senior high group, mix group, and university group. The results suggested this variable had significant effects on the math anxiety-performance link (*Q* = 11.65, *p* = 0.02). More specifically, the senior high group had the largest math anxiety-performance link (*r* = −0.44), followed by the junior high group (*r* = −0.39), university (*r* = −0.33), elementary (*r* = −0.27), and mixed group, which had the smallest math anxiety-performance link (*r* = −0.21).

### Publication Years

**Figure 4:**
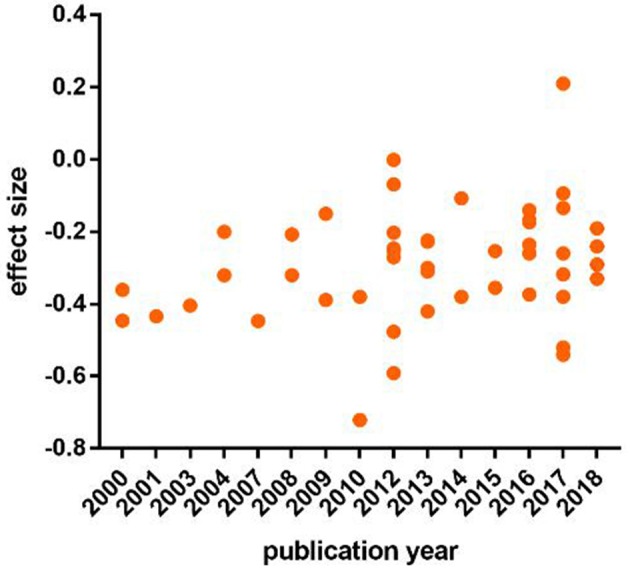
Relationship between publication year and effect size.

The current meta-analysis contained the time frame of 19 years (2000–2018). To evaluate whether time had an effect on the math anxiety-performance link, a Pearson correlation was conducted between the publication years and the of all individual studies (*r* = 0.27, *p* > 0.05). No correlation was found between effect sizes and the publication years (Figure 4).

### Measurement of MA

We also explored whether the scales used to test math anxiety had an effect on the math anxiety-performance link. The results suggested that the measurement of MA had no effect on the math anxiety-performance link (*Q* = 6.54, *p* = 0.48). Specifically, individual studies that used other scales (e.g., the Mathematics Anxiety Scale for Elementary School Students [MASESS; Bindak, 2005] had the largest math anxiety-performance link (*r* = −0.41), followed by studies that used the MAS (*r* = −0.40), MARS (*r* = −0.35), MAQ (*r* = −0.28), MASYC (*r* = −0.26), CMAQ (*r* = −0.24), SEMA (*r* = −0.24), and AMAS (*r* = −0.24), and individual studies that used the MASC had the smallest math anxiety-performance link (*r* = −0.15).

### Measurement of Math Performance

The classification criteria used to code the “math performance” variable are different. In terms of the testing aspects of the math performance test, two skills were highlighted: calculation and problem solving. In this study, the measurement aspects of the test had significant effects on the math anxiety-performance link (*Q* = 17.001, *p* < 0.01). Studies that evaluated mixed math performance (calculation and problem solving) reported the largest effects (*r* = −0.37). In addition, it is worth noting that studies in which the test assessed problem-solving skills in math performance reported larger effects (*r* = −0.33), while studies that assessed the calculation ability in math performance reported smaller effects (*r* = −0.21). In terms of the form of the math performance test, three types of math performance tests were identified: custom test, standardized test and others. There was a significant difference among these conditions (*Q* = 13.02, *p* = 0.001). The results suggested that studies that adopted a standardized test custom test reported larger effects (*r* = −0.33) than studies that adopted a standardized test (*r* = −0.28). Moreover, the studies that adopted other math tests reported the largest effects (*r* = −0.51).

## Discussion

In summary, this meta-analysis illustrated a significantly negative effect size in the math anxiety-performance association. Furthermore, six moderator variables that were assumed have effect on the association between MA and math performance were tested in the present study. The size of the links differed across geographical regions, grade level, measurement of MA, measurement forms of math performance and measurement aspects of math performance.

### The Significant Correlation Between MA and Math Performance

With a significantly negative effect size (*r* = −0.32), the result was consistent with previous individual studies (Miller and Bichsel, 2004; Rodarte-Luna and Sherry, 2008) and was also consistent with meta studies in general (Hembree, 1990; Ma, 1999). To further interpret the results of the meta-analysis, we compared our results with mainly two previous meta-analytic reviews (Hembree, 1990; Ma, 1999) and other studies, which examined the reciprocal relationship between MA and math achievement.

First, as the meta-analysis by Ma (1999) was limited to individual studies conducted from 1978 to 1992 across elementary to senior high schools, it can be argued that our investigation has expanded and extended Ma's (1999) study, in terms of the time period (2000–2018 vs. 1978–1992) and grade level (elementary- senior high school vs. elementary-university). In addition, Ma (1999) found a smaller effect size (*r* = −0.27) than the effect size in our meta-analysis study (*r* = −0.32). Similarly, in another meta-analysis that explored the construct of MA, the meta-analysis by Hembree (1990) did not focus on the math anxiety-performance association, although some analyses were conducted on this topic. Thus, our study updated the findings in terms of the time period (2000–2018 vs. before 1990). Furthermore, Hembree (1990) reported a greater effect size (*r* = −0.31) than Ma's (1999) studies, although it was smaller than the effect size that we found in this study. Our results are also consistent with other individual studies (Reali et al., 2016; Justicia-Galiano et al., 2017; Lauer et al., 2018). For example, Reali et al. (2016) explored the relationship between MA and math performance among Colombian students and obtained a negative correlation (r = −0.27); this result was also found among children aged 8 to 12 years (Justicia-Galiano et al., 2017). Thus, the effect size in our study implied a robust negative math anxiety-performance link.

To explain this negative link, two theories were posed. The Deficit Theory claims that poor performance in a math test would lead to higher anxiety and uncomfortable experiences in the future. That is, bad math performance would deficit the willingness to study math and lead to MA (Berch and Mazzocco, 2007; Carey et al., 2016). Moreover, the debilitating anxiety model suggested that MA would influence math performance by cognitive interference. For example, individuals who have higher levels of MA frequently avoid involving math learning. Thus, they have less chance to practice their math learning skills (Ashcraft, 2002; Carey et al., 2016). Overall, these two theories explained why negative correlation between MA and math performance existed.

### Moderation Effects

Regarding the specific factors that modulate the math anxiety-performance association, we identified 6 modulate variables. The results differed across geographical regions, grade level, measurement of MA, measurement forms of math performance and measurement aspects of math performance.

### Moderating Role of Geographical Regions

The results indicated that the relationship significantly differed among geographical regions. Specifically, the math anxiety-performance relationship was suggested to be the strongest in the studies that involved Asian students and was the second strongest in the studies that involved US students, whereas it was the weakest in the studies that involved European students, which was partly consistent with previous studies (Ching, 2017; Wu et al., 2017). This finding also extended the previous meta-analyses by Ma (1999) and Hembree (1990). Ma (1999) did not provide details regarding whether geographical regions (US vs. Europe vs. Asia vs. other regions) modulated the math anxiety-performance association. Similarly, Hembree (1990) also did not include this factor in his analysis. Furthermore, for Asian students, the more significantly robust negative math anxiety-performance association indicated that the more anxiety they experience in their math learning, the worse learning performance they would achieve.

To our knowledge, in Asian, teachers and parents all highly valued academic achievement of their students (children). For some parents, they even according to grades to predict whether their children would be success in the future. Moreover, there were stiff competitions in school. For example, students would experience entrance examinations as the first step of their formal schooling (Ho et al., 2000). Thus, understandably, Asian students always compared their own rankings with rankings of other students and overvalued academic achievements. furthermore, to meet their goals, Asian students always pushed them hard. Once they cannot achieve their goals, they would experience anxiety and disappointed in themselves (Whang and Hancock, 1994). As a result, the negative math anxiety-performance association of Asian students might attribute to these factors.

Furthermore, in the Western European countries where not overemphasized the relationship between academic achievement and success, students did not overvalue their academic performance. And they often experience relaxed math learning environment. For students who originated from the United States, although they highlighted the significance of learning math, students seem to experience less anxiety.

### Moderating Role of Grade Level

In terms of grade level, we found significant differences in the math anxiety-performance link. Specifically, students from senior high school showed the highest negative math anxiety-performance correlation, and students from elementary school showed the lowest negative math anxiety-performance correlation. This result was somewhat different from the findings in Ma's (1999) study, which suggested no grade differences in the math anxiety-performance relationship (for the Grades 4 through 6 vs. Grades 10 through 12 comparison; for the Grades 7 through 9 vs. Grades 10 through 12 comparison). In contrast, this result was in line with recent studies that suggested the negative math-performance relationship surfaced in secondary education (Ashcraft and Krause, 2007; Maloney and Beilock, 2012; Hill et al., 2016). It is understandable that this link develops stronger in the high school educational period. During the higher educational period, students are exposed to more difficult math curriculum, while more cognitive engagement is acquired. Furthermore, as the onset of adolescence, this stage was important for students to develop social and emotional regulation skills. These changes might undoubtedly influence how students engage in math, which, in turn, was likely to affect their emotional reaction toward the subject.

### Moderating Role of the Measurement of MA

We found the measurement of MA modulates the math anxiety-performance link, which could explain, in part, why research found variable correlations between MA and math performance (Dowker et al., 2012; Wood et al., 2012; Haase et al., 2012; Wu et al., 2012, 2014). Specifically, the individual studies that applied other questionnaires (e.g., the Mathematics Anxiety Scale for Elementary School Students (MASESS; Bindak, 2005) to test the MA reported the highest negative correlation. First, although all measurements exhibited good reliability, some individual studies extracted the items from different MA scales and combined them to assess the MA. Second, it is suggested that the aspects of MA that were estimated by these questionnaires were somewhat different from each other. For example, we found that some studies measured whether MA related to the inability to successfully solve math questions (Krinzinger et al., 2007, 2009; Dowker et al., 2012; Wood et al., 2012; Haase et al., 2012). Interestingly, no math anxiety-performance link was detected in these studies. Other studies explored anxiety in math-related situations (Wu et al., 2012, 2014; Ramirez et al., 2013). In these studies, the participants did not experience the failure of math question-solving and were only instructed to evaluate the level of anxious they experienced when they calculate mathematical questions or when they were in mathematical situations.

### Moderating Role of the Measurement of Math Performance

First, consistent with prior studies (Ramirez et al., 2016; Lee and Cho, 2018), we found the measurement aspects of math performance modulated the math anxiety-performance link, with a stronger negative correlation in studies that evaluated problem solving skills than in studies that evaluated basic calculation skills. Compared with a complex problem-solving test, the basic calculation was easier and occupied less cognitive resources. Individuals would solve these problems with a more relaxed mood and experience less frustration; thus, they would have better math performance. Second, the measurement forms of math performance were also shown to modulate the negative math anxiety-performance link, with a stronger negative correlation in studies that adopted custom tests than in studies that adopted standard tests. The custom tests were used to assess specific aspects of math and the test items were extracted from more than one test file (Ashkenazi and Danan, 2017), while standard tests were used to assess the general ability of math with standard evaluation criteria. The difficulty level of custom tests might be difficult to control; however, the difficulty level of standard tests had been assessed very well.

### Moderating Role of Gender

This study indicated there were no significant gender differences in the math anxiety- achievement link, although prior research found a significantly greater level of MA in females than in males (Ho et al., 2000; Osborne, 2001; Yüksel-Sahin, 2008). In our studies, only 7 studies provided the raw data (correlation value and sample size) for a meta-analysis of the math anxiety- achievement link in different genders. Thus, this result should be interpreted with caution. First, this finding is consistent with Ma's (1999) finding, which suggests no differences in the math anxiety-performance link for boys and girls. Furthermore, the results that suggested gender less affected the math performance is in line with studies that showed no gender differences or less gender difference in gender equal countries (Spelke, 2005; Guiso et al., 2008; Elsequest et al., 2010; Goetz et al., 2013). Finally, previous studies suggested there were gender differences in MA. Specifically, girls reported more MA than boys (Devine et al., 2012). However, there is no direct evidence that indicates gender modulated the math anxiety-performance link. Taken together, it seems no gender differences in the math anxiety-performance relationship existed.

### Moderating Role of Publication Year

This study also reported there is no significant effect of publication year. The studies that found no negative math anxiety-performance may be attributed to other factors. It is conceivable that a non-significant difference was identified in publication years, which suggested that the negative math anxiety-performance relationship is robust among recent 19 years. Although educators have proposed alleviating the negative emotion toward the MA, it is still a long way to go.

## Limitations and Implications

Despite the novel findings and implications of this study, several limitations should be clarified. First, only 7 of the 49 studies included measured the gender difference in the math anxiety-performance link, while the other studies all reported the math anxiety-performance link based on a mixed gender sample. Thus, as mentioned above, the interpretation of the lack of a moderate effect should proceed with caution.

Second, although the present study obtained studies conducted in multiple countries (i.e., China, Turkey and England), these studies were all reported by English. If we considered the studies written in other languages, our findings would be enrich in this field.

Furthermore, to develop a better understanding regarding the math anxiety-achievement link, additional details (e.g., moderator analysis) could be given for a range of math performance tests (e.g., computation, logical reasoning, or concept understanding). Unfortunately, limited by the levels of a moderator variable and restricted by the number of studies included in this meta-analysis, we did not perform a more detailed analysis separately for test types of math performance.

Therefore, future studies could extend further to explore the following questions. Moreover, some implications based on our results can be applied in an educational context. First, to develop a better understanding of the effect of gender on the math anxiety-performance link, more individual studies should be included in future studies. Although a range of previous studies explored the relationship between gender and MA and tended to support that girls suffered more anxiety in the math study context, the evidence suggested that gender did not modulate the relationship between MA and math performance (Hembree, 1990; Ma, 1999; Devine et al., 2012; Wu et al., 2012). Thus, to explore strong evidence for a correlation between gender and the math anxiety-performance link, additional researches should be conducted; these explorations would help researchers and educators to adopt eligible methods to relieve the MA and promote the math performance of students. For female students, girls are often told that boys would have a better performance than girls, which would pose a threat to their attitude towards math learning (Beasley and Fischer, 2012). Thus, one way to relieve their MA is to eliminate stereotype threats toward them regarding their math learning and encourage them in the math learning context. For example, encouraging female students when they solve a problem successfully rather than consoling them by saying, “It's OK girls cannot solve this problem.”, which may pose a stereotype threat for them. Second, according to our results, the strongest math anxiety-performance link was found among senior high school students. Thus, the mitigation of the MA of students in senior high school is essential. For educators in senior high school, increasing the difficulty of learning materials step by step to prevent students from feeling anxious regarding mathematics learning is necessary. Thus, students would have a solid mathematical knowledge base. This rule in line with previous evidence, which suggested that a solid foundation in mathematical knowledge is beneficial for relieving MA and improving math performance (Beilock and Willingham, 2014). Finally, our results suggest different effects of the measurement of math performance on the math anxiety-performance link. Thus, for educators, they should consider the characteristics of each type of mathematical knowledge and design corresponding lessons. Using this approach, students would adapt effortlessly to the progress of the math course.

## Conclusion

The present study, which included a meta-analysis of 49 studies, indicated a robust negative math anxiety-performance correlation, and this relationship was moderated by geographical region, grade level, measurement of MA, measurement aspects of math performance and measurement forms of math performance. The math anxiety-performance link was stronger among Asian students than among European/American students. Moreover, this negative link was strongest among senior high school students. Finally, this negative link was strongest among studies using custom tests and studies that assessed problem-solving skills.

## Author Contributions

JZ provided the idea, designed this study, contributed to data collection, the manuscript writing, and paper revise. NZ designed this study, contributed to data collection, the manuscript writing, and paper revise. QK provided the idea, contributed to study design, data analysis and paper revise. All authors approval of the version to be published and agree to be accountable for all aspects of the work.

### Conflict of Interest Statement

The authors declare that the research was conducted in the absence of any commercial or financial relationships that could be construed as a potential conflict of interest.
